# Mechanical Properties of Rock Salt from the Kłodawa Salt Dome—A Statistical Analysis of Geomechanical Data

**DOI:** 10.3390/ma17143564

**Published:** 2024-07-18

**Authors:** Malwina Kolano, Marek Cała, Agnieszka Stopkowicz

**Affiliations:** Faculty of Civil Engineering and Resource Management, AGH University of Krakow, 30-059 Krakow, Poland; cala@agh.edu.pl (M.C.); agnieszka.stopkowicz@agh.edu.pl (A.S.)

**Keywords:** rock salt, Kłodawa diapir, uniaxial compressive strength, Young’s modulus, Poisson’s ratio, tensile strength, cohesion, internal friction angle

## Abstract

Rock salt is a potential medium for underground storage of energy resources and radioactive substances due to its physical and mechanical properties, distinguishing it from other rock media. Designing storage facilities that ensure stability, tightness, and safety requires understanding the geomechanical properties of rock salt. Despite numerous research efforts on the behaviour of rock salt mass, many cases still show unfavourable phenomena occurring within it. Therefore, the formulation of strength criteria in a three-dimensional stress state and the prediction of deformation processes significantly impact the functionality of storage in salt caverns. This article presents rock salt’s mechanical properties from the Kłodawa salt dome and a statistical analysis of the determined geomechanical data. The analysis is divided into individual mining fields (Fields 1–6). The analysis of numerical parameter values obtained in uniaxial compression tests for rock salt from mining Fields 1–6 indicates an average variation in their strength and deformation properties. Upon comparing the results of Young’s modulus (E) with uniaxial compressive strength (UCS), its value was observed with a decrease in uniaxial compressive strength (E = 4.19968·UCS^2^, R-square = −0.61). The tensile strength of rock salt from mining Fields 1–6 also exhibits moderate variability. An increasing trend in tensile strength was observed with increased bulk density (σ_t_ = 0.0027697·ρ − 4.5892, r = 0.60). However, the results of triaxial tests indicated that within the entire range of normal stresses, the process of increasing maximum shear stresses occurs linearly ((σ_1_ − σ_3_)/2 = ((σ_1_ + σ_3_)/2)·0.610676 + 2.28335, r = 0.92). A linear relationship was also obtained for failure stresses as a function of radial stresses (σ_1_ = σ_3_·2.51861 + 32.9488, r = 0.73). Based on the results, the most homogeneous rock salt was from Field 2 and Field 6, while the most variable rock salt was from Field 3.

## 1. Introduction

Rock salt is considered one of the best alternatives for underground storage of energy resources [1,2,3,4,5,6,7]. Salt formations are widely used for storing oil and natural gas [8,9,10] and are also considered for storing radioactive substances [8,11,12]. Increasing attention is being given to salt caverns for their potential use in underground storage of hydrogen and compressed air [13,14,15,16,17,18].

Rock salt is highly regarded as a medium for underground storage due to its physical and mechanical properties. It is a rock with low porosity and permeability (10^−21^–10^−24^ m^2^), possessing good mechanical properties such as self-healing of damage, favourable creep properties, plasticity, and relatively low strength [19,20,21,22,23,24,25,26,27,28,29,30,31].

Since the durability and reliability of storing energy resources and waste are crucial, conducting thorough investigations of the rock salt mass serving as the storage medium is essential. Although underground storage facilities are generally safer and more stable than their above-ground counterparts, poor design and usage can lead to serious accidents [9,26]. Therefore, the safety and stability of underground storage are among the most important criteria in the geomechanical design process.

The specific geological structure of salt domes significantly influences the design and construction of underground storage facilities. The considerable variation in internal structure means that large underground facilities are accompanied by salt series with variable properties, including geomechanical characteristics [32]. Moreover, long-term operation can lead to significant deformations and even loss of storage volume due to changes in internal pressures and the time-dependent behaviour of rock salt [33]. Additionally, using storage facilities changes the conditions in the surrounding rock mass. The stress state around salt caverns depends not only on their depth and the in situ stress state of the rock mass but also on the internal pressure of the stored resource [25,26,34]. In the case of gas or air storage, this can lead to cyclic changes in the stress state.

Despite numerous research efforts on the behaviour of the rock salt mass, many cases still exhibit adverse phenomena that threaten the proper functioning of these facilities. Examples include volume shrinkage, excessive convergence, overburden settlement, increased permeability of the rock mass, migration of the stored substance, and even the collapse of storage caverns [1,32,35,36]. These issues indicate that the problem of developing effective methods for designing underground storage remains unresolved.

Formulating strength criteria in a three-dimensional stress state and predicting deformation processes significantly impact the functionality of storage facilities. The properties of the rock salt mass distinguish it from other rock media. So far, laboratory studies indicate its unique characteristics compared to other rock materials [32,37,38,39,40,41,42,43,44,45,46,47]. Due to the plastic nature of the salt medium, determining its fundamental mechanical properties and behaviour is complex and challenging to define accurately. Salt exhibits elastic and inelastic behaviour as a function of stress magnitude, conditions, and loading time [24,35,48,49,50,51].

A natural property of rock salt is its heterogeneity. Therefore, it is impossible to formulate quantitative conclusions about the behaviour of designed underground structures based on a fragmentary sampling of the deposit [46,52,53,54,55]. It is important to consider that the variability in salt series structures, their geological history, and the current depth of deposition can significantly influence the diversity of geomechanical properties. Geomechanical phenomena occurring in the rock salt mass surrounding underground excavations are predicted using basic strength and deformation parameters of elastic rocks. The primary source of information on the mechanical properties of the rock mass comes from laboratory tests, such as uniaxial compression tests (compressive strength, modulus of elasticity, Poisson’s ratio), triaxial compression tests, uniaxial tensile tests or the Brazilian method, and creep tests under uniaxial constant load (salt viscosity).

Increasingly, determining rock parameters involves directly observing the rock mass behaviour or verifying numerical modelling results under in situ measurement conditions [53,56,57]. In situ tests are mainly limited to convergence measurements of excavations [58,59].

The most popular rock strength indicator is uniaxial compressive strength (UCS). Rock deformation properties are typically described using two parameters: the modulus of elasticity (E) and Poisson’s ratio (υ). Since brittle failure of rock material occurs when the ultimate shear strength and the ultimate tensile strength are exceeded, tensile strength is also an important strength parameter for rocks. Alongside compression tests, tensile tests form the basis of studies on the mechanical properties of materials [53,55]. Due to the difficulties in obtaining core material and preparing laboratory samples of sufficient length for direct tensile tests, the tensile strength is often determined using the Brazilian test method, which involves diametral compression of cylindrical samples.

The strength and deformation properties of rocks determined under uniaxial compression or tension are insufficient for practical applications. Therefore, additional studies are conducted to determine the rock strength in a three-dimensional stress state (triaxial compression). The primary objective of triaxial compression tests is to formulate a strength criterion, which is difficult for rock salt. Analysis of test results from salt samples from the Mogilno dome and the Sieroszowice deposit indicates that linear strength conditions can only be applied up to a certain low-stress level [32,60].

## 2. Geological Settings

**Figure 1:**
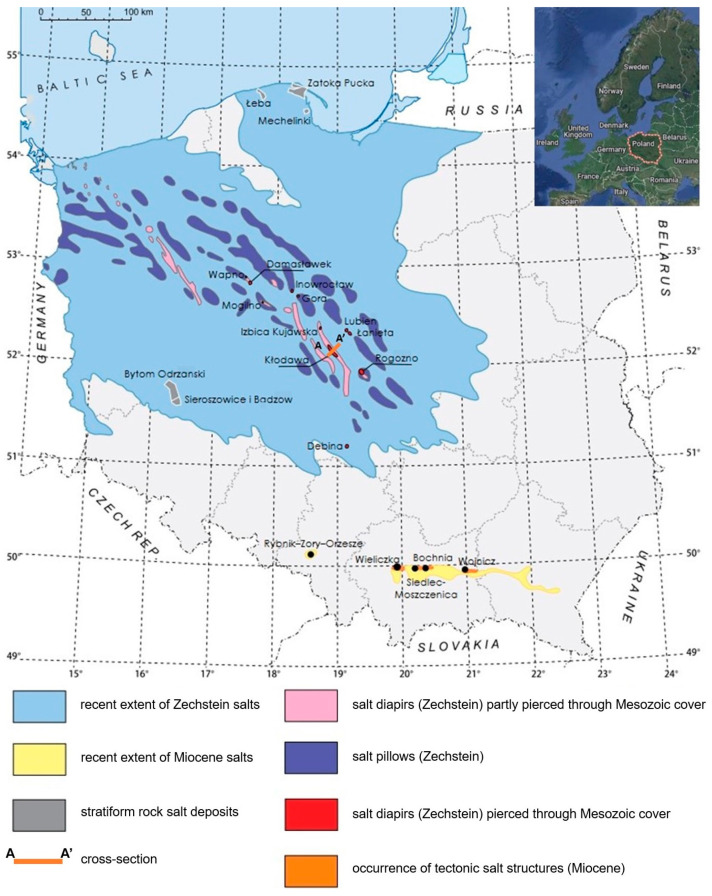
Map of rock salt deposits in Poland [64].

**Figure 2:**
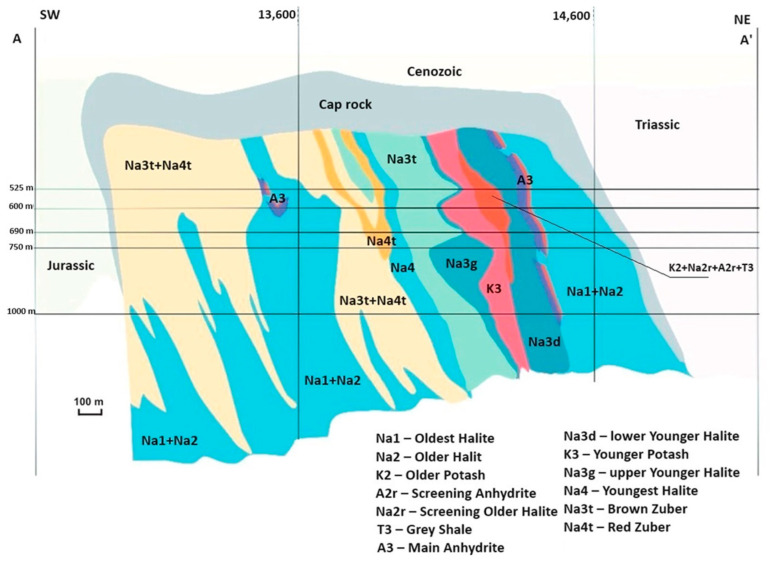
Cross-section of the Kłodawa salt dome [65].

The Kłodawa salt deposit is located in central Poland, within the Izbica Kujawsko-Łęczycka salt structure, on the western edge of the Kujawy Ridge (Figure 1). This deposit formed during the Zechstein period, approximately 200–250 million years ago. It is a subsurface feature covered by Tertiary and Quaternary sediments, which form a longitudinal anticlinal structure (Figure 2). Within the core of this anticline are deposits of Zechstein salt-bearing formations, which migrate upwards through a strongly arched Mesozoic formation. The Kłodawa salt deposit stretches from northwest to southeast for approximately 26 km. Its width at the upper part varies from 0.5 to 2 km [61,62,63]. It is the largest salt deposit in the Polish Lowlands.

The salt deposit is covered by a layer of clayey gypsum cap with variable thicknesses ranging from 50 to 300 m (Figure 2). In the central part, above the clayey gypsum cap, there are transgressive Neogene formations, while in the southwestern and northeastern parts, the salt deposit is covered by rocks of older Mesozoic, mainly composed of breccias [66,67]. The salt deposit is bounded by Triassic and Jurassic formations from the northeastern side. Conversely, from the southwestern side, Jurassic formations adjoin it (Figure 2). The internal structure of the salt deposit is heavily folded, distinguishing two extreme anticlinal forms separated by a deep syncline. The core of the anticline consists of older salts (Na2), while younger salts (Na3) are present on the southwestern side. The anticline along the southwestern boundary of the deposit is formed by older salts (Na2) and the oldest (Na1). Meanwhile, the syncline between the marginal anticlines is composed of pink rock salt (Na4) [67].

**Figure 3:**
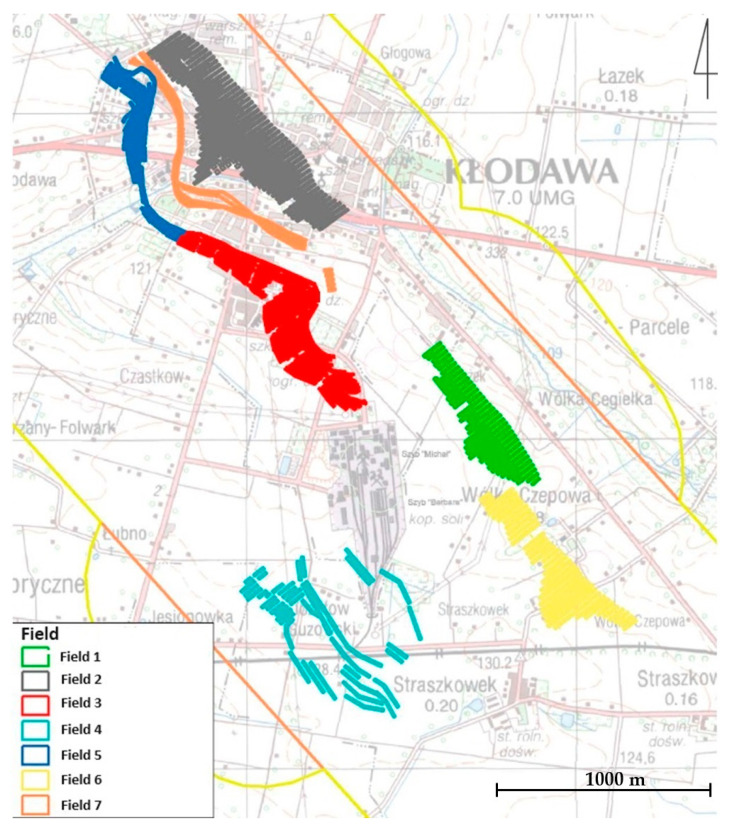
Map of the exploitation fields [67].

Currently, there are seven mining fields (Field 1–Field 7) and twelve mining levels (450, 475, 500, 525, 550, 575, 600, 630, 660, 690, 720, and 750) at depths ranging from 322 to 625 m below sea level (Figure 3). The levels within the mining fields are further divided into sublevels of 25 m or 30 m in height.

The mining fields are located in the central part of the deposit and cover an area approximately 8 km in length (according to the strike direction) and 2 km in width, consisting of the following sites [67]:

- Field 1—mining on the five shallowest levels;
- Field 2—mining on levels corresponding to relative depths of 450–600 m below sea level (m b.s.l.);
- Field 3—mining between levels corresponding to relative depths of 450 and 750 m b.s.l.;
- Field 4—mining on levels corresponding to relative depths of 538, 572, and 600 m b.s.l.;
- Field 5—mining between levels corresponding to relative depths of 600 and 750 m b.s.l.;
- Field 6—exploration with galleries;
- Field 7—mining on levels corresponding to relative depths of 575 and 600 m b.s.l., consisting of potassium–magnesium salts.

Exploitation of the Kłodawa salt deposit is forecasted until 2052 [68]. The development of rock salt extraction is planned at deeper levels in all mining fields. In mining Fields 2, 3, 5, and 7, mining is anticipated to depths of 657–698 m b.s.l. (mining levels 780 and 820) [68].

## 3. Materials and Methods

### 3.1. Test Materials

**Figure 4:**
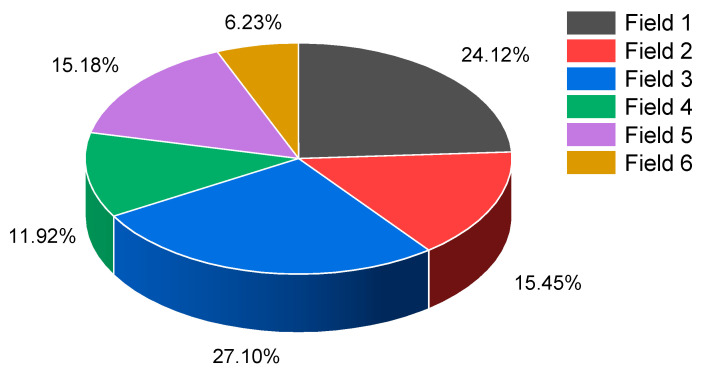
Percentage list of tested samples depending on the mining field.

Materials obtained from chambers of Fields 1, 2, 3, 4, 5, and 6 underwent testing. However, the material from Field 7 was excluded from the analysis due to its potassium–magnesium salt content. In total, 369 samples of rock salt were tested and subjected to statistical evaluation (Field 1—89, Field 2—57, Field 3—100, Field 4—44, Field 5—56, and Field 6—23) (Figure 4).

All samples (rectangular prisms) were taken from freshly exposed underground extraction fields to exclude the impact of weathering on the analysed material. The relative depths of the extracted rectangular prisms range from 475 to 780 m below sea level. Cylindrical samples (diameter ≈ 5 cm, height ≈ 10/5/2.5 cm) were prepared from cuboidal samples (approximately 11 cm × 12 cm × 12 cm, Table 1) using dry rolling or diamond coring methods. The prepared samples were evaluated for correctness of execution by measuring them with an accuracy of 0.01 mm. The dimensions of the samples comply with ISRM (International Society for Rock Mechanics) recommendations [69].

The samples were used to conduct the following tests: uniaxial compression test (UCT), triaxial compression test (TCT), and Brazilian tensile test (splitting tensile strength test) (BT). Table 2 presents the number of tested samples and their utilisation.

### 3.2. Test Methods

#### 3.2.1. The Determination of Physical Properties

The volumetric density was determined according to ISRM guidelines [69] for each prepared sample using the following formula:

(1) $$\rho =\frac{M}{V}$$

where

M—mass of the sample determined with an accuracy of 0.01 g;

V—volume of the sample.

The dimensions were measured with an accuracy of 0.01 mm.

To determine the approximate porosity of the analysed rock salt, it was assumed that it is fully composed of halite with a density ρ_s_ = 2.16 g/cm^3^ [70] and that its volumetric density (ρ) is equal to the volumetric density of the skeleton (ρ_d_). With this assumption, the porosity n can be expressed by the following formula:

(2) $$n=1-\frac{\rho }{\rho_{s}}$$

The porosity determined in this way does not account for inclusions and impurities present in the rock salt.

#### 3.2.2. Uniaxial Compressive Strength Test

A hydraulic press with automatic piston feed and force recording was used for the uniaxial compression strength tests. The tests were conducted at a constant loading rate. Axial, radial, and volumetric displacements were recorded with an accuracy of 0.01 mm during the tests.

The uniaxial compression strength (σ_c_) was determined based on the ratio of the maximum destructive force (P_max_), which was obtained during uniaxial compression of the salt sample in the press to the cross-sectional area (A_0_):

(3) $$\sigma_{c}=\frac{P_{max}}{A_{0}}$$

Based on the stress–strain characteristics, the strain coefficients were determined as follows:

- Longitudinal strain coefficient (E) calculated from the average slopes of the more-or-less straight line portion of the axial stress–axial strain curve (~0.2 σ_c_–~0.8 σ_c_) (Average Modulus, ISRM [69]):
  (4) $$E=\frac{\Delta \sigma }{\Delta \varepsilon_{z}}$$
- Lateral strain coefficient (ν) within the linearity range of radial strains:

(5) $$\nu =-\frac{\Delta \varepsilon_{r}}{\Delta \varepsilon_{z}}$$

where

Δσ—axial stress change;

Δε_z_—axial strain produced by the stress change;

Δε_r_—diametric strain produced by the stress change.

#### 3.2.3. Tensile Strength Test

Due to the difficulties in obtaining core material and preparing laboratory samples of sufficient length for direct tensile testing, the Brazilian test method was used to determine the compressive strength. In this method, a cylindrical sample is loaded by compressive force along opposite faces.

Tensile testing using the Brazilian test method was conducted using a strength testing machine.

The tensile strength (σ_t_) was determined according to the following formula:

(6) $$\sigma_{t}=\frac{0.636\cdot P}{D\cdot t}$$

where

P—load at failure;

D—diameter of the test specimen;

t—thickness of the test specimen measured at the centre.

#### 3.2.4. Triaxial Compressive Strength Test

**Figure 5:**
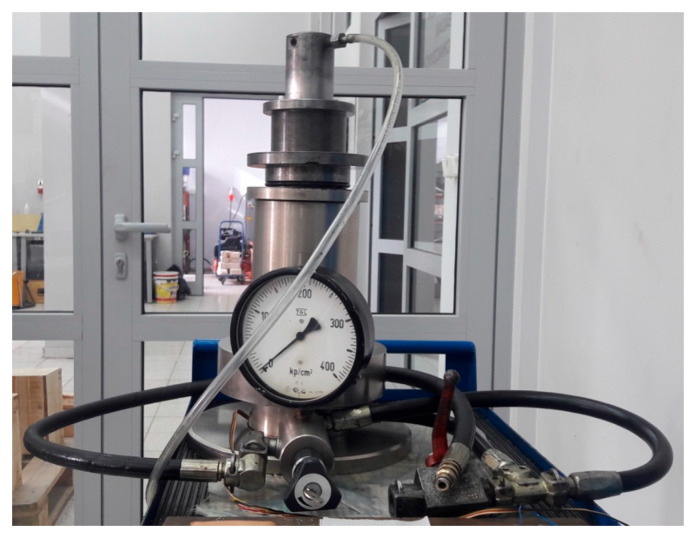
High-pressure chamber of the Karman type.

Triaxial compression tests were conducted in a hydraulic press equipped with a pressure chamber to maintain the desired pressure constant throughout the test (Figure 5), operating in a constant axial strain rate control mode. During the test, the axial strains were recorded as a function of the applied axial loads, with constant radial stress values.

The triaxial compression tests were conducted at radial pressures ranging from approximately 2 MPa to 25 MPa and according to test type I (individual test, ISRM [69]). Several tests were conducted to determine individual points on the failure (peak strength) envelope.

To formulate the strength criterion for the investigated rock salt, the Coulomb–Mohr criterion was utilised, which assumes a linear form of stress function:

(7) $$\left(\sigma_{1}-\sigma_{3}\right)/2-\left((\sigma_{1}+\sigma_{3}\right)/2)\cdot \sin\phi -c\cdot \cos\phi =0$$

where

σ_1_—maximum axial stress;

σ_3_—confining pressure;

c—cohesion;

φ—internal friction angle.

Moving on to the envelope equation, we obtained the classical form of the Coulomb criterion:

(8) τ=c+σ·tgφ

which, on the stress plane (σ, τ), represents a linear Mohr circle envelope.

## 4. Results and Discussion

### 4.1. Physical Property

**Figure 6:**
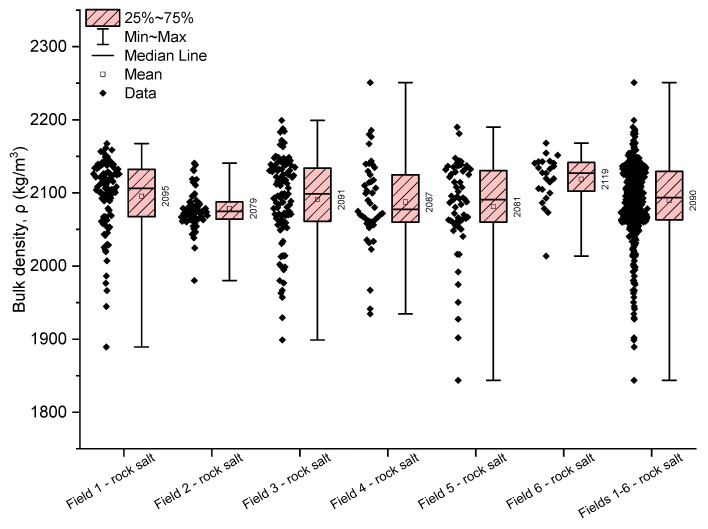
The rock salt bulk density results for Fields 1–6.

The volumetric density of rock salt from the Kłodawa deposit ranges from 1844 to 2251 kg/m^3^ (with an average value of 2090 kg/m^3^ and a median of 2093 kg/m^3^), exhibiting very low variability (with a mean coefficient of variation of 2.55%). The highest average volumetric density is found in the rock salt from Field 6 (2119 kg/m^3^), while the lowest is in the rock salt from Field 2 (2079 kg/m^3^) (Table 3, Figure 6).

The slight variations in the analysed characteristic indicate the homogeneity of the rock salt from Fields 1–6 in terms of volumetric density.

**Figure 7:**
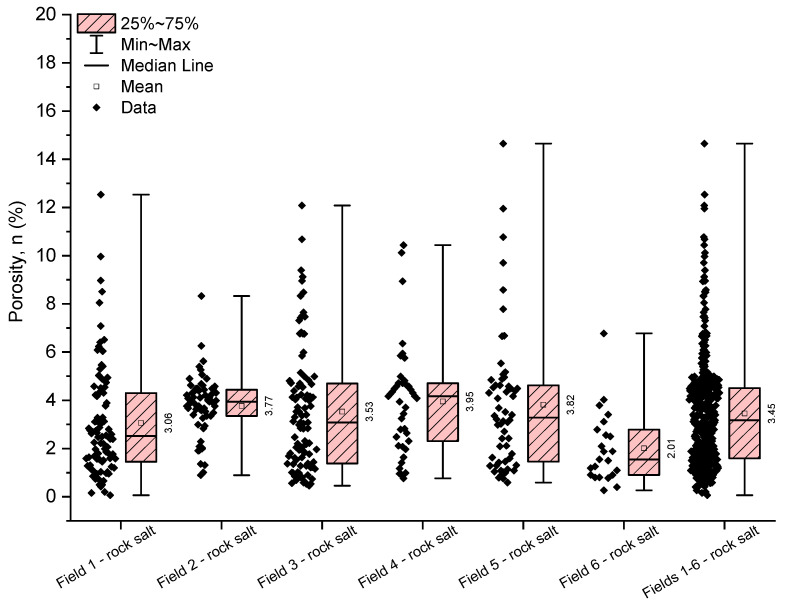
The rock salt porosity results for Fields 1–6.

The estimated porosity determined for the rock salt from Fields 1–6 exhibits strong variability, ranging from 0.06% to 14.65% (Table 4, Figure 7). Referring to the average porosity value of 3.45%, it can be concluded that the rock salt from the Kłodawa deposit is a material with low porosity. The highest average porosity of 3.95% (with a median of 4.17%) is characteristic of the rock salt from Field 4, while the highest porosity was exhibited by the rock salt from Field 6 (with an average porosity of 2.01% and a median of 1.54%). The analysed material in terms of porosity is highly diverse (with a coefficient of variation of 67.60% for rock salt from Fields 1–6).

However, it should be noted that the presented porosity values are only approximate and do not account for inclusions and impurities in the analysed rock salt (with the specific density equal to the density of halite).

### 4.2. Uniaxial Compressive Strength and Deformability of Material

**Figure 8:**
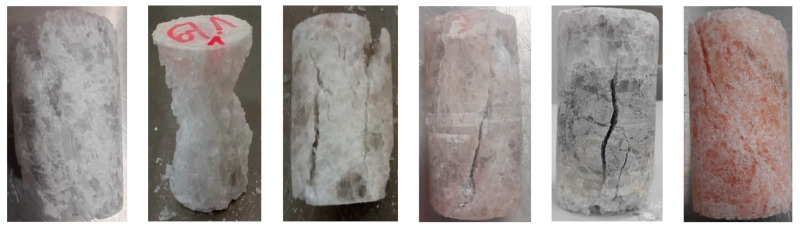
Examples of failure of samples—UCT.

During the uniaxial tests, the rock salt samples underwent dynamic, brittle failure either at the maximum load or upon transitioning into the critical phase. Failure occurred by sliding along the shear surface or complete fragmentation of the sample (Figure 8).

**Figure 9:**
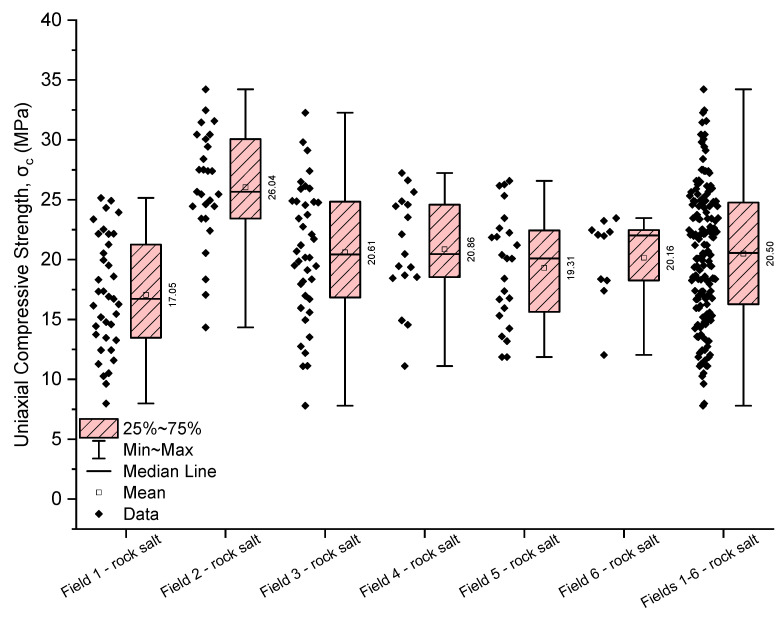
The rock salt uniaxial compressive strength results for Fields 1–6.

**Figure 10:**
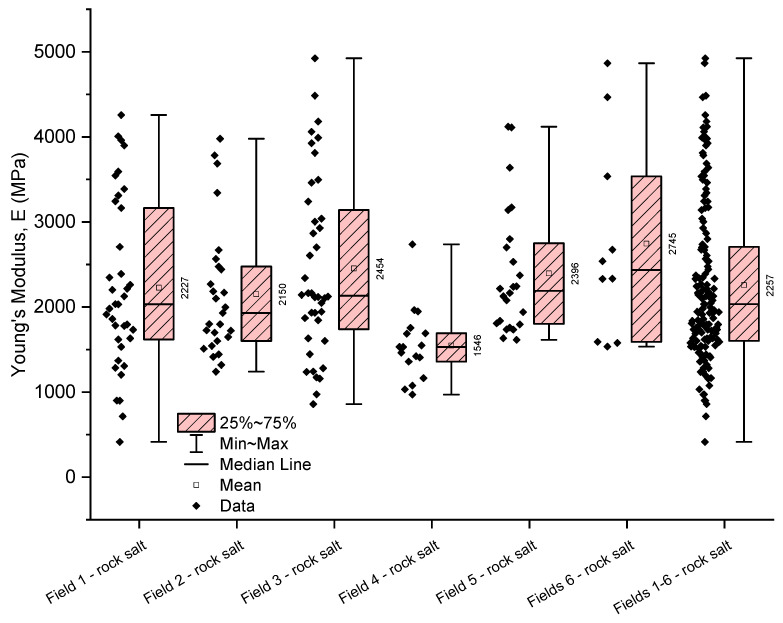
The rock salt Young’s modulus results for Fields 1–6.

**Figure 11:**
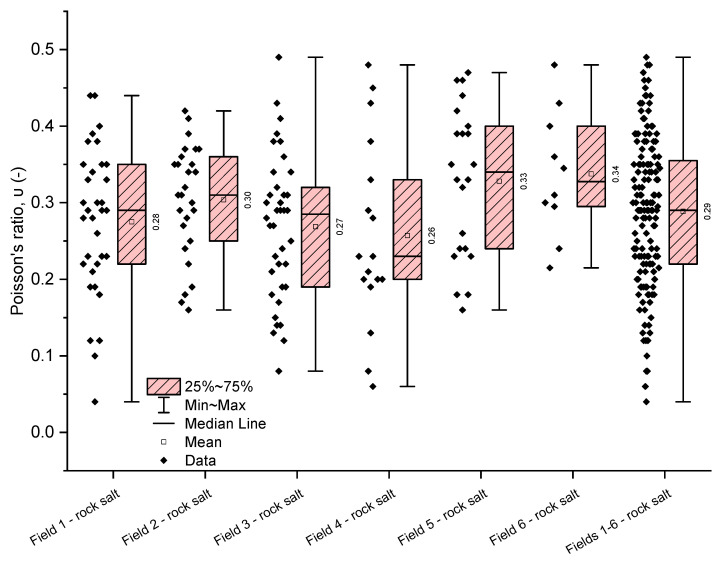
The rock salt Poisson’s ratio results for Fields 1–6.

The analysis of numerical parameters obtained in uniaxial compression tests for all examined rock salt samples from Fields 1–6 indicates average variability in their strength and deformation properties (Table 5, Table 6 and Table 7, Figure 9, Figure 10 and Figure 11).

The uniaxial compressive strength of the rock salt samples from Fields 1–6 ranges from 7.81 to 34.22 MPa (with an average value of 20.50 MPa and a median of 20.55 MPa), with a coefficient of variation of 27.42%, indicating average variability (Table 5, Figure 9). The greatest variability in uniaxial compressive strength (average variability) characterises the rock salt from Field 1, with an average strength value of 17.05 MPa (coefficient of variation 27.61%) (Table 5). On the other hand, rock salt from Fields 6 (coefficient of variation 17.02%, average strength 20.16 MPa) and 2 (coefficient of variation 17.97%, average strength 26.04 MPa) exhibits low variability in uniaxial compressive strength (Table 5). Analysing the obtained values of uniaxial compressive strength, it can be concluded that the highest average strength was observed in the rock salt from Field 2 (average strength of 26.04 MPa), while the lowest is in the rock salt from Field 1 (average strength of 17.05 MPa) (Table 5).

The deformation parameters of the analysed rock salt from the Kłodawa deposit exhibit greater variability than uniaxial compressive strength values. However, they still show average variability.

The obtained values of Young’s modulus for the rock salt from Fields 1–6 range from 414 to 4923 MPa (with an average value of 2326 MPa and a median of 2123 MPa), with a coefficient of variation close to 45% (coefficient of variation 42.12%) (Table 6, Figure 10). The greatest variability in Young’s modulus characterises the rock salt from Field 1 (coefficient of variation 44.55%), with Young’s modulus values ranging from 414 to 4257 MPa (average value of 2227 MPa, median 2032 MPa). On the other hand, the smallest variation in Young’s modulus values was observed in the rock salt from Field 4 (971–2736 MPa, average value of 1546 MPa, median 1531 MPa). The lowest average Young’s modulus was obtained for the rock salt from Field 4 (average value of 1546 MPa, median 1531 MPa), while the highest is for the rock salt from Field 3 (average value of 2454 MPa, median 2133 MPa).

The Poisson’s ratio for the rock salt from Fields 1–6 ranges from 0.04 to 0.49, with a mean value of 0.29 (median 0.29) (Table 7, Figure 11). The obtained values exhibit average variability (coefficient of variation 33.60%). The least variability in the Poisson’s ratio was observed in the rock salt from Fields 6 (coefficient of variation 23.30%) and 2 (coefficient of variation 23.78%). For these fields, the Poisson’s ratio shows low variability. The highest variability was observed in the rock salt from Field 4, where the Poisson’s ratio ranges from 0.06 to 0.48 (mean value 0.26, median 0.23), with a coefficient of variation of 46.52%. This rock salt is heterogeneous regarding the obtained values of the Poisson’s ratio (strongly varied).

**Figure 12:**
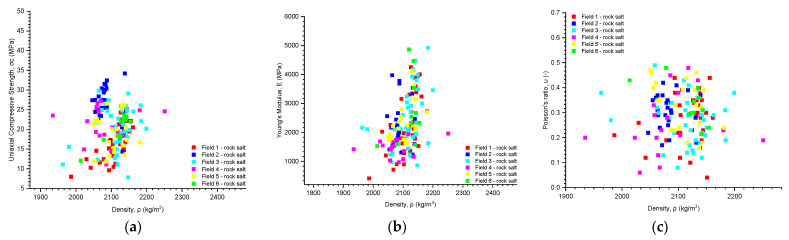
The dependence of (**a**) uniaxial compressive strength; (**b**) Young’s modulus; and (**c**) Poisson’s ratio on density.

**Figure 13:**
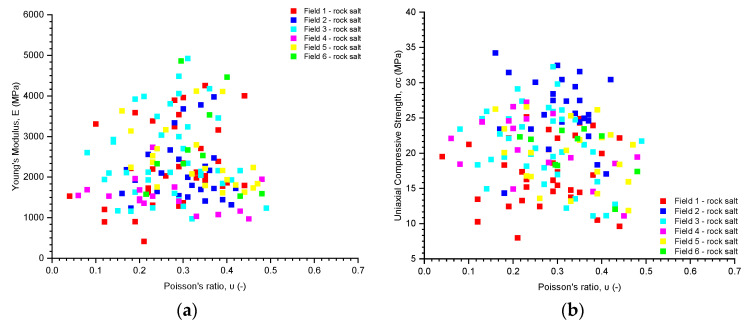
The dependence of (**a**) Young’s modulus and (**b**) uniaxial compressive strength on Poisson’s ratio.

After comparing the parameters obtained from the uniaxial compression test, namely the uniaxial compressive strength, Young’s modulus, and Poisson’s ratio, with bulk density, no mutual dependencies were observed (Figure 12). Additionally, no dependencies were found when comparing Young’s modulus and uniaxial compressive strength with Poisson’s ratio (Figure 13).

**Figure 14:**
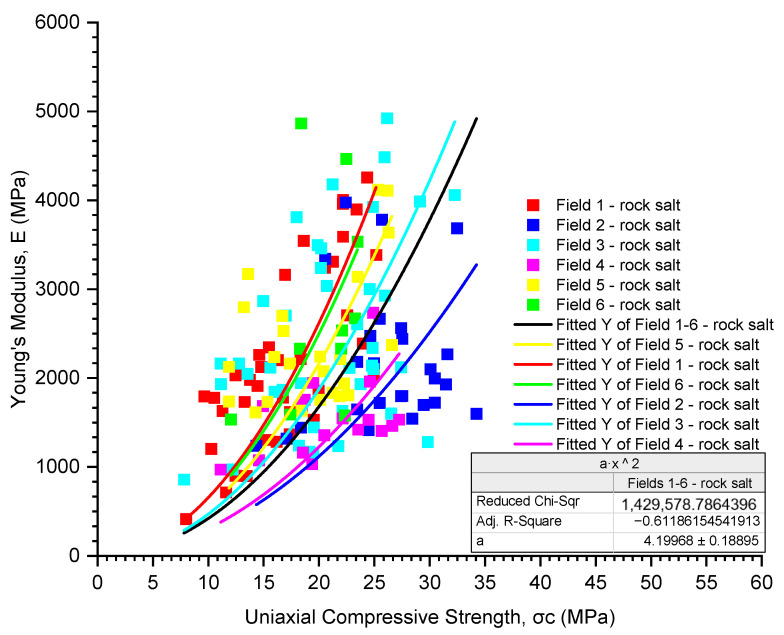
The dependence of Young’s modulus on uniaxial compressive strength (E-UCS).

However, considering the obtained results of Young’s modulus as a function of uniaxial compressive strength (UCS), a trend of decreasing Young’s modulus (E) with decreasing UCS can be observed (Table 8, Figure 14). For all tested samples from Fields 1–6, a quadratic relationship was observed (E = 4.19968·UCS^2^), with a coefficient of determination R-square of −0.61. Analysing the relationship between Young’s modulus and uniaxial compressive strength for the individual fields of the rock salt, the highest coefficient of determination was obtained for the rock salt from Field 4 (E = 3.06393·UCS2, R-square = −0.87). However, the fitting model for the rock salt from Field 1 and Field 3 is unsatisfactory (R-square of 0.39 and −0.31, respectively), and for the samples from Field 6, the coefficient of determination is close to 0, indicating no relationship between the variables.

### 4.3. Indirect Tensile Strength

**Figure 15:**
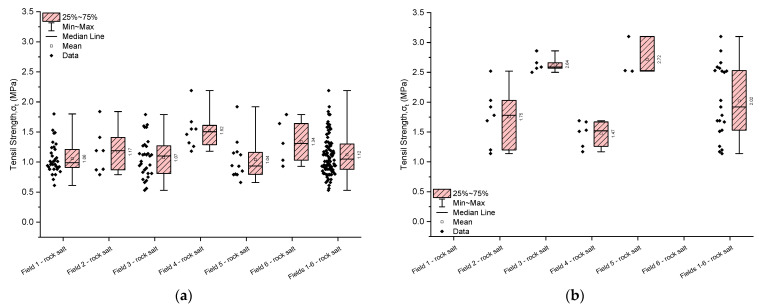
The rock salt tensile strength results for Fields 1–6. (**a**) Height-to-diameter ratio equals ~0.51; (**b**) Height-to-diameter ratio equals ~1.02.

The cross-cutting tests were conducted on samples with different slenderness ratios (~0.51 and ~1.02); hence, the results in Table 9 and Figure 15 are presented accordingly. Additionally, considering that ISRM guidelines recommend a slenderness ratio of approximately ~0.5 for the tested samples, the results were analysed for samples with a slenderness ratio of ~0.5.

The tensile strength of the rock salt from Fields 1–6 ranges from 0.53 to 2.19 MPa (with a mean value of 1.12 MPa and a median of 1.05 MPa) and exhibits moderate variability (coefficient of variation 29.49%) (Table 9, Figure 15). The most homogeneous material in terms of tensile strength, with the highest average tensile strength, is the rock salt from Field 4, with values ranging from 1.18 to 2.19 MPa (mean value of 1.52 MPa and median of 1.51 MPa). On the other hand, the most heterogeneous material is the rock salt from Field 5, which exhibits average variability and the lowest average tensile strength (mean value of 1.04 MPa and median of 0.94 MPa). Its tensile strength values range from 0.66 to 1.92 MPa.

**Figure 16:**
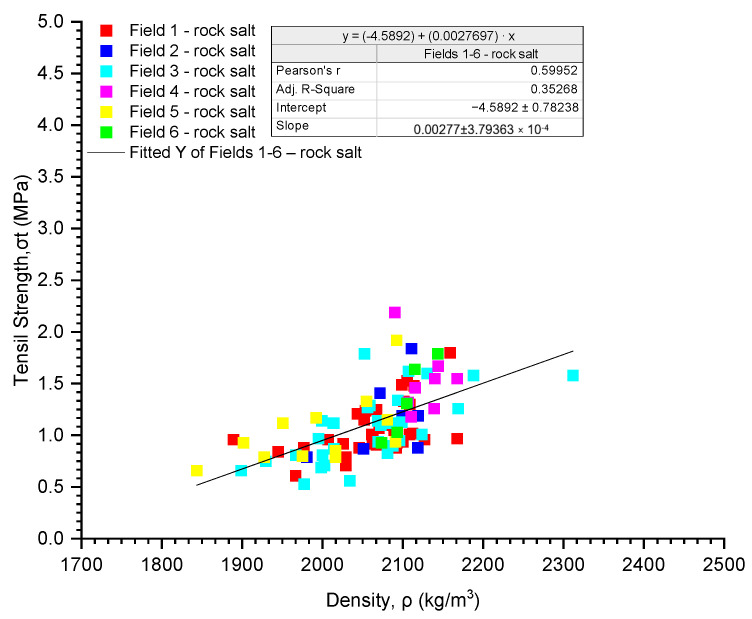
The dependence of tensile strength on density (σ_t_-ρ).

Analysing the influence of bulk density on the tensile strength of the analysed rock salt from Fields 1–6 (samples with an aspect ratio of ~0.51, combined dataset), a tendency of increasing tensile strength with increasing bulk density was observed (Figure 16). There is a linear relationship with moderate correlation strength (σ_t_ = 0.0027697·ρ − 4.5892, r = 0.60) (Table 10). The strongest correlation between tensile strength and bulk density was observed in rock salt from Field 6 (σ_t_ = 0.0135688·ρ − 27.2324, r = 0.95), (σ_t_ = −0.00301262·ρ + 7.92826, r = −0.24, weak correlation).

### 4.4. Strength of Materials in Triaxial Compression

In triaxial tests, the compressive strength was determined as the ratio of the maximum force at which sample failure or rapid axial deformation occurred at the cross-sectional area of the sample.

**Figure 17:**
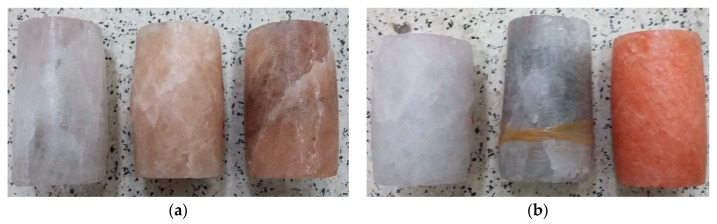
Examples of sample failure—TCT: (**a**) σ_3_ = 5 MPa; (**b**) σ_3_ = 10 MPa.

**Figure 18:**
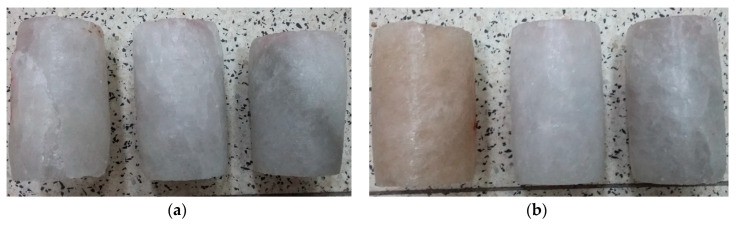
Examples of sample failure—TCT: (**a**) σ_3_ = 15 MPa; (**b**) σ_3_ = 20 MPa.

In triaxial tests, at lower radial stresses (similarly to uniaxial tests), samples experienced brittle failure at maximum load or after transitioning to the critical phase, with failure occurring in the form of sliding along the shear surface. At higher stress levels, samples underwent significant deformation while retaining partial cohesion. Examples of sample failure are illustrated in Figure 17 and Figure 18.

**Figure 19:**
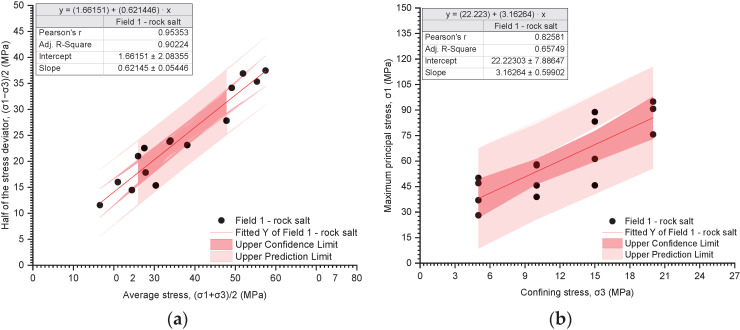
The results of triaxial tests for Field 1. (**a**) The stress path (σ_1_ − σ_3_)/2 − (σ_1_ + σ_3_)/2; (**b**) the influence of radial pressure (σ_3_) on the value of failure stress (σ_1_).

**Figure 20:**
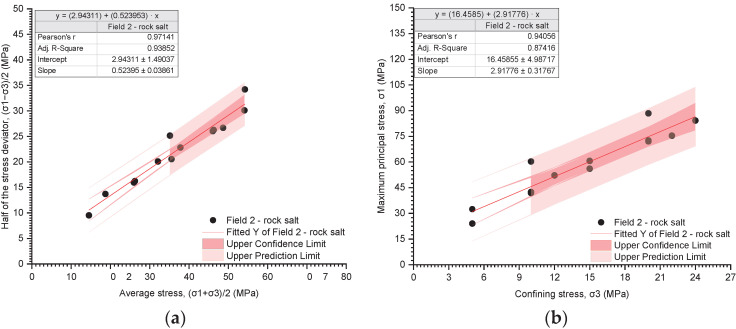
The results of triaxial tests for Field 2. (**a**) The stress path (σ_1_ − σ_3_)/2 − (σ_1_ + σ_3_)/2; (**b**) the influence of radial pressure (σ_3_) on the value of failure stress (σ_1_).

**Figure 21:**
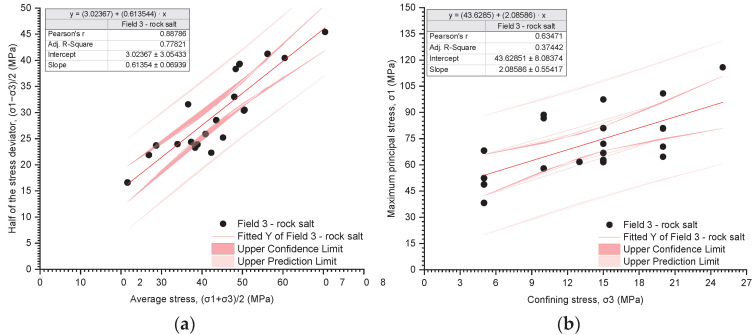
The results of triaxial tests for Field 3. (**a**) The stress path (σ_1_ − σ_3_)/2 − (σ_1_ + σ_3_)/2; (**b**) the influence of radial pressure (σ_3_) on the value of failure stress (σ_1_).

**Figure 22:**
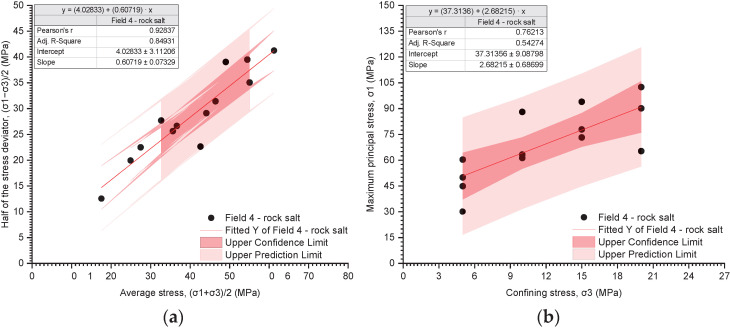
The results of triaxial tests for Field 4. (**a**) The stress path (σ_1_ − σ_3_)/2 − (σ_1_ + σ_3_)/2; (**b**) the influence of radial pressure (σ_3_) on the value of failure stress (σ_1_).

**Figure 23:**
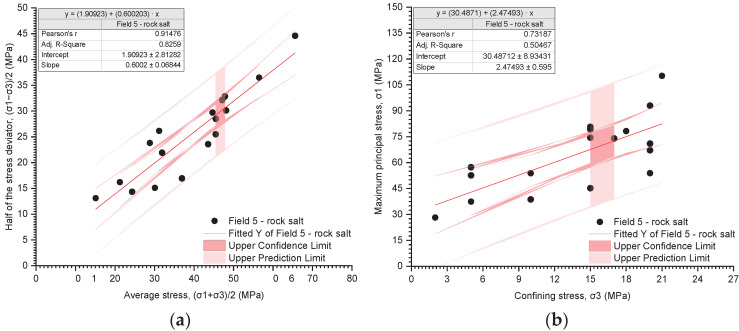
The results of triaxial tests for Field 5. (**a**) The stress path (σ_1_ − σ_3_)/2 − (σ_1_ + σ_3_)/2; (**b**) the influence of radial pressure (σ_3_) on the value of failure stress (σ_1_).

**Figure 24:**
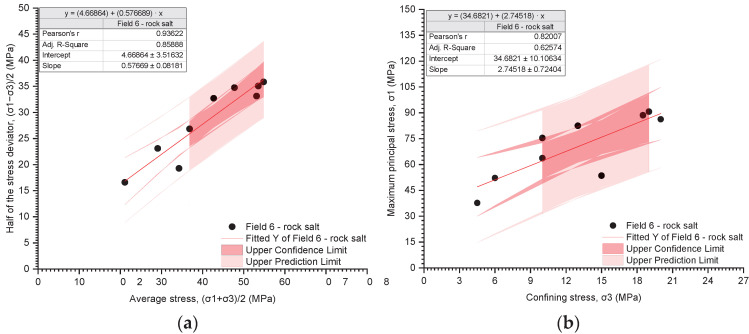
The results of triaxial tests for Field 6. (**a**) The stress path (σ_1_ − σ_3_)/2 − (σ_1_ + σ_3_)/2; (**b**) the influence of radial pressure (σ_3_) on the value of failure stress (σ_1_).

**Figure 25:**
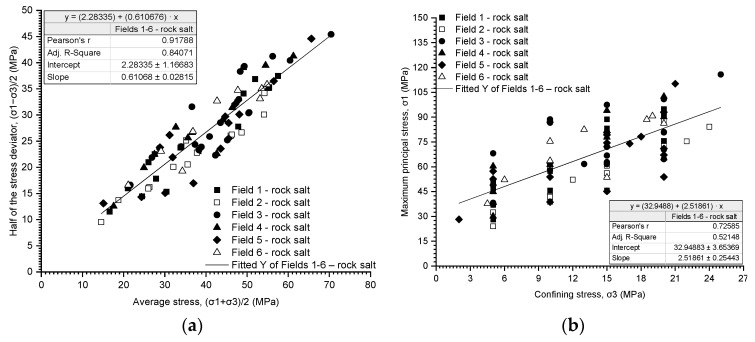
The results of triaxial tests for Fields 1–6. (**a**) The stress path (σ_1_ − σ_3_)/2 − (σ_1_ + σ_3_)/2; (**b**) the influence of radial pressure (σ_3_) on the value of failure stress (σ_1_).

The results of the triaxial tests are presented utilising the stress path ((σ_1_ − σ_3_)/2 − (σ_1_ + σ_3_)/2) and the relationship between the deviatoric stress and the radial stress (σ_1_ − σ_3_) (Figure 19, Figure 20, Figure 21, Figure 22, Figure 23, Figure 24 and Figure 25).

The analysis of the stress path ((σ_1_ − σ_3_)/2 − (σ_1_ + σ_3_)/2) indicates that over the entire range of normal stresses ((σ_1_ + σ_3_)/2), the process of increasing maximum shear stresses ((σ_1_ − σ_3_)/2) occurs linearly (Figure 19a, Figure 20a, Figure 21a, Figure 22a, Figure 23a, Figure 24a and Figure 25a). For all rock salt samples from Fields 1–6 combined, the following relationship was obtained: (σ_1_ − σ_3_)/2 = ((σ_1_ + σ_3_)/2)٠0.610676 + 2.28335, characterised by a very strong correlation (r = 0.92) (Table 11). Analysing the results for rock salt from individual fields, the best correlation between normal and shear stresses was obtained for rock salt from Field 2 (r = 0.97, very strong correlation), while the lowest was observed for rock salt from Field 3 (r = 0.89, moderately strong correlation) (Table 11).

The results of the triaxial compression tests also indicate a linear relationship between the ultimate stresses (σ_1_) and the radial stresses (σ_3_) over the entire pressure range at which individual tests were conducted (Figure 19b, Figure 20b, Figure 21b, Figure 22b, Figure 23b, Figure 24b and Figure 25b). However, this relationship exhibits a lower correlation strength than the stress path (specifically, r = 0.73, r = 0.92).

Overall, for all samples from Fields 1–6, a reasonably strong linear relationship between ultimate stresses and radial stresses was obtained, expressed as σ_1_ = σ_3_·2.51861 + 32.9488 (r = 0.73) (Table 12). Analysing the results obtained for materials from individual fields, the lowest correlation between ultimate stresses and radial stresses was exhibited by rock salt from Field 3 (r = 0.63), while the highest correlation was observed for rock salt from Field 2 (r = 0.94) (Table 12).

For the tested samples, within the entire range of radial stresses, the equilibrium equation can be formulated in the form of the classic Coulomb criterion with a linear envelope and the following parameters (Table 13):

- Field 1–6—stress path, Pearson’s r = 0.91788;
- Cohesion: 2.88 MPa;
- Internal friction angle: 37.64°;
- Fields 1–6—critical state, Pearson’s r = 0.72585;
- Cohesion: 10.38 MPa;
- Internal friction angle: 25.57°.

Since the strength parameters (c and φ) were determined with greater accuracy from the stress path, the results obtained from this relationship were considered for further analysis.

The cohesion of rock salt from Fields 1–6 ranges from 2.12 to 5.72 MPa, with the highest value obtained for rock salt from Field 6 and the lowest for rock salt from Field 1. Meanwhile, the angle of internal friction of rock salt from Fields 1–6 ranges from 31.60° to 38.42°. The highest values were obtained for rock salt from Field 1 and the lowest from Field 2.

The equation τ=2.88+σ·tg(37.64) adequately describes the strength condition of rock salt within the range of analysed radial pressures (~2–25 MPa).

## 5. Conclusions

Rock salt from the Kłodawa deposit, originating from mining Fields 1–6, is homogeneous in terms of volumetric density but highly variable in terms of porosity.

The analysis of numerical parameters obtained from uniaxial compression tests for all examined rock salt samples from mining Fields 1–6 indicates average variability in their strength and deformation properties. Additionally, the deformation parameters of the analysed rock salt from the Kłodawa deposit exhibit greater variability than the values of uniaxial compression strength.

After comparing the parameters obtained in the uniaxial compression test, namely uniaxial compression strength, Young’s modulus, and Poisson’s ratio, with volumetric density, no mutual dependencies were observed. Similarly, no dependencies were found when comparing Young’s modulus and uniaxial compression strength with Poisson’s ratio.

Considering the obtained results of Young’s modulus as a function of uniaxial compression strength, a trend of decreasing Young’s modulus (E) with decreasing uniaxial compression strength (UCS) can be observed. The overall analysis of all examined samples from mining Fields 1–6 revealed a quadratic dependence of Young’s modulus on uniaxial compression strength (E = 4.19968·UCS^2^, R-square = −0.61).

The tensile strength of rock salt from mining Fields 1–6 is characterised by average variability (coefficient of variation 29.49%). Analysing the influence of volumetric density on the tensile strength of the analysed rock salt from Fields 1–6 (samples with a slenderness ratio of ~0.51, combined dataset), a trend of increasing tensile strength with increasing volumetric density was observed. There is a linear relationship with moderate correlation strength (σ_t_ = 0.0027697·ρ − 4.5892, r = 0.60).

The results of triaxial tests indicate that within the entire range of normal stresses ((σ_1_ + σ_3_)/2), the process of increasing maximum shear stresses ((σ_1_ − σ_3_)/2) occurs linearly. For all samples of rock salt from mining Fields 1–6 combined, the following relationship was obtained: (σ_1_ − σ_3_)/2 = ((σ_1_ + σ_3_)/2)·0.610676 + 2.28335, characterised by a very strong correlation (r = 0.92).

The results of triaxial compression tests also indicate a linear relationship between the failure stresses (σ_1_) and the radial stresses (σ_3_) throughout the entire range of pressures at which individual tests were conducted. However, this relationship exhibits a lower correlation strength than the stress path (r = 0.73, r = 0.92).

For the tested samples, within the entire range of radial stresses, the equilibrium equation can be formulated in the form of the classical Coulomb’s law with a linear envelope and the following parameters:

- Fields 1–6—stress path, Pearson’s r = 0.91788: τ=2.88+σ·tg(37.64);
- Fields 1–6—critical state, Pearson’s r = 0.72585: $\tau =10.38+\sigma \cdot tg\left(25.57\right).$

In summary, the results showed that rock salt from Fields 2 and 6 was the most homogeneous, while rock salt from Field 3 exhibited the highest variability (porosity—strength variation, UCS—average variation, E—average variation, ν—average variation, and TS—average variation). The overall analysis of the obtained results for rock salt from mining Fields 1–6 indicates an average level of variation (Table 14). To identify the factors influencing the level of variability in the analysed geomechanical parameters of rock salt from the Kłodawa salt deposit, further analyses of the obtained results about the depth of the collected samples are planned.

Properties of rock mechanics of rock salt are critical input data for numerical models used in the design, implementation, and operation stages of caverns and risk assessment. Therefore, it is extremely important to use representative values for various properties of rock salt. The results presented here, obtained based on research conducted on a large research sample, which includes division into mining fields and statistical analysis, can be successfully used in modelling underground storage facilities for energy resources. It should also be noted that the analysed results were obtained in the laboratory from tests on small samples (compared to the entire rock mass). Therefore, the results may be affected by a phenomenon known as the scale effect. However, to fully illustrate the strength–deformation behaviour of the rock salt from the Kłodawa deposit and to model its behaviour during storage, it is necessary to analyse its dilatancy and creep behaviour. Achieving a stress state that causes the opening of microcracks and the creation of new ones can change rock salt’s permeability from practically non-existent to measurable. After the opening of microcracks, percolation pathways are activated [71], leading to hydraulic behaviours that cannot be ignored. Additionally, understanding the geomechanical behaviour of rock salt under cyclic and dynamic loading and via simulation of repetitive gas filling and emptying is an important aspect. Moreover, the rock joints in rock salt are very important for the engineering of underground storage of energy resources and radioactive substances. The lithostratigraphic units of the Kłodawa salt deposit are intensely folded internally and dip very steeply, but they do not exhibit fractures. Investigating the strength parameters at the contact points of individual lithostratigraphic units would be valuable too, but obtaining such samples for testing is very challenging [72,73,74].

## Figures and Tables

**Table 1:** Examples of the test materials and the prepared cylindrical samples.

**Field 1**		
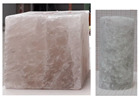	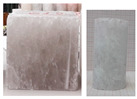	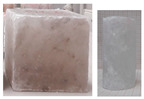
**Field 2**		
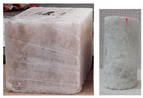	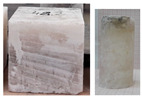	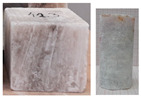
**Field 3**		
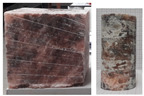	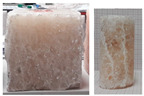	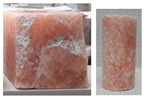
**Field 4**		
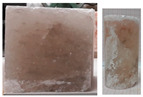	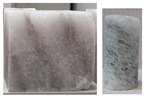	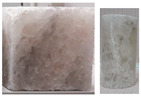
**Field 5**		
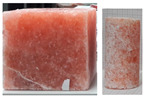	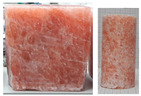	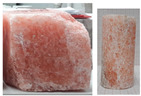
**Field 6**		
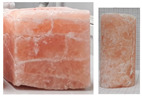	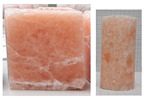	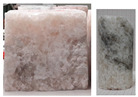

**Table 2:** Number of samples tested and their utilisation in the experiments.

Field	Type of Test ^1^	Number of Samples	Height-to-Diameter Ratio [-]		
			Average	Min	Max
Field 1	UCT	37	2.01	1.82	2.05
	BT	34	0.51	0.50	0.55
	TCT	18	2.02	2.00	2.03
Field 2	UCT	27	2.02	2.00	2.04
	BT	16	0.80	0.49	1.03
	TCT	14	1.84	1.56	2.04
Field 3	UCT	40	2.00	1.56	2.03
	BT	36	0.59	0.48	1.07
	TCT	24	2.01	1.95	2.04
Field 4	UCT	17	2.00	1.77	2.13
	BT	14	0.71	0.49	1.07
	TCT	13	2.03	1.98	2.11
Field 5	UCT	24	2.02	1.96	2.07
	BT	15	0.61	0.51	1.02
	TCT	17	2.02	1.98	2.05
Field 6	UCT	10	1.97	1.84	2.02
	BT	5	0.52	0.51	0.52
	TCT	8	2.00	1.96	2.03

^1^ UCT—uniaxial compression test; BT—Brazil test; and TCT—triaxial compression test.

**Table 3:** Summary of bulk density values.

Number of Samples	Average [kg/m^3^]	Median [kg/m^3^]	Min [kg/m^3^]	Max [kg/m^3^]	Standard Dev. [kg/m^3^]	Coeff. of Variation [%]
Field 1						
89	2095	2106	1889	2167	50	2.41
Field 2						
57	2079	2075	1980	2141	27	1.31
Field 3						
100	2091	2099	1899	2199	59	2.82
Field 4						
44	2087	2077	1935	2251	59	2.84
Field 5						
56	2081	2091	1844	2190	64	3.07
Field 6						
23	2119	2127	2014	2168	33	1.54
			Fields 1–6			
369	2090	2093	1844	2251	53	2.55

**Table 4:** Summary of porosity values.

Number of Samples	Average [%]	Median [%]	Min [%]	Max [%]	Standard Dev. [%]	Coeff. of Variation [%]
Field 1						
87	3.06	2.52	0.06	12.54	2.31	75.43
Field 2						
57	3.77	3.94	0.89	8.33	1.26	33.39
Field 3						
89	3.53	3.08	0.45	12.09	2.57	72.77
Field 4						
39	3.95	4.17	0.76	10.44	2.23	56.50
Field 5						
54	3.82	3.28	0.58	14.65	2.87	75.20
Field 6						
22	2.01	1.54	0.26	6.78	1.47	72.90
Fields 1–6						
348	3.45	3.18	0.06	14.65	2.33	67.60

**Table 5:** Summary of uniaxial compressive strength (UCS) values.

Number of Samples	Average [MPa]	Median [MPa]	Min [MPa]	Max [MPa]	Standard Dev. [MPa]	Coeff. of Variation [%]
Field 1						
37	17.05	16.73	7.98	25.15	4.71	27.61
Field 2						
27	26.04	25.67	14.34	34.22	4.68	17.97
Field 3						
40	20.61	20.44	7.81	32.26	5.55	26.93
Field 4						
17	20.86	20.47	11.11	27.23	4.46	21.39
Field 5						
24	19.31	20.10	11.86	26.58	4.53	23.45
Field 6						
10	20.16	22.03	12.04	23.47	3.43	17.02
			Fields 1–6			
155	20.50	20.55	7.81	34.22	5.62	27.42

**Table 6:** Summary of Young’s modulus (E) values.

Number of Samples	Average [MPa]	Median [MPa]	Min [MPa]	Max [MPa]	Standard Dev. [MPa]	Coeff. of Variation [%]
Field 1						
37	2227	2032	414	4257	992.23	44.55
Field 2						
27	2150	1930	1240	3978	750.79	34.92
Field 3						
40	2454	2133	859	4923	1039.34	42.35
Field 4						
17	1546	1531	971	2736	411.28	26.60
Field 5						
24	2396	2190	1615	4120	732.85	30.58
Field 6						
10	2745	2436	1535	4866	1124.00	40.95
			Fields 1–6			
155	2326	2123	414	4923	979.81	42.12

**Table 7:** Summary of Poisson’s ratio (ν) values.

Number of Samples	Average [-]	Median [-]	Min [-]	Max [-]	Standard Dev. [-]	Coeff. of Variation [%]
Field 1						
37	0.28	0.29	0.04	0.44	0.10	34.67
Field 2						
27	0.30	0.31	0.16	0.42	0.07	23.78
Field 3						
40	0.27	0.29	0.08	0.49	0.09	34.66
Field 4						
17	0.26	0.23	0.06	0.48	0.12	46.52
Field 5						
24	0.33	0.34	0.16	0.47	0.10	29.42
Field 6						
10	0.34	0.33	0.22	0.48	0.08	23.30
			Fields 1–6			
155	0.29	0.29	0.04	0.49	0.10	33.60

**Table 8:** The results of the regression analysis for E-UCS.

Number of Samples	Equation	R-Square	Standard Error
Field 1			
37	E = 6.55213٠UCS^2^	0.38995	0.36606
Field 2			
27	E = 2.79638٠UCS^2^	−0.65720	0.25702
Field 3			
40	E = 4.69291٠UCS^2^	−0.31149	0.37505
Field 4			
17	E = 3.06393٠UCS^2^	−0.87374	0.28790
Field 5			
24	E = 5.40696٠UCS^2^	−0.59085	0.44740
Field 6			
10	E = 6.25274٠UCS^2^	−0.0653	0.88573
Fields 1–6			
155	E = 4.19968٠UCS^2^	−0.61186	0.18895

**Table 9:** Summary of tensile strength values.

Height-to-Diameter Ratio [-]	Number of Samples	Average [MPa]	Median [MPa]	Min [MPa]	Max [MPa]	Standard Dev. [MPa]	Coeff. of Variation [%]
Field 1							
0.51	34	1.06	0.99	0.61	1.80	0.25	23.63
Field 2							
0.51	7	1.17	1.19	0.79	1.84	0.34	29.43
1.02	9	1.75	1.78	1.14	2.52	0.44	25.3
Field 3							
0.51	31	1.07	1.10	0.53	1.79	0.32	30.21
1.05	5	2.64	2.59	2.50	2.86	0.12	4.67
Field 4							
0.51	8	1.52	1.51	1.18	2.19	0.30	19.43
0.99	6	1.47	1.52	1.17	1.69	0.19	13.24
Field 5							
0.51	12	1.04	0.94	0.66	1.92	0.33	31.47
1.01	3	2.72	2.53	2.52	3.10	-	-
Field 6							
0.51	5	1.34	1.31	0.93	1.79	0.33	24.92
Fields 1–6 (2–5)							
0.51	97	1.12	1.05	0.53	2.19	0.33	29.49
1.02	23	2.02	1.92	1.14	3.10	0.60	29.68

**Table 10:** The results of regression analysis for σ_t_-ρ.

Number of Samples	Equation	Pearson’s r	R-Square
Field 1			
34	σ_t_ = 0.00230718·ρ − 3.70222	0.53117	0.2597
Field 2			
7	σ_t_ = 0.00363454·ρ − 6.38697	0.49255	0.09112
Field 3			
31	σ_t_ = 0.00270609·ρ − 4.49716	0.66000	0.41614
Field 4			
8	σ_t_ = −0.00301262·ρ + 7.92826	−0.23861	−0.10024
Field 5			
12	σ_t_ = 0.00259767·ρ − 4.1447	0.60291	0.29985
Field 6			
5	σ_t_ = 0.0135688·ρ − 27.2324	0.94963	0.86907
Fields 1–6			
97	σ_t_ = 0.0027697·ρ − 4.5892	0.59952	0.35268

**Table 11:** The results of regression analysis for stress path ((σ_1_ − σ_3_)/2 − (σ_1_ + σ_3_)/2).

Number of Samples	Equation	Pearson’s r	R-Square
Field 1			
18	(σ_1_ − σ_3_)/2 = ((σ_1_ + σ_3_)/2)·0.621446 + 1.66151	0.95353	0.90224
Field 2			
14	(σ_1_ − σ_3_)/2 = ((σ_1_ + σ_3_)/2)·0.523953 + 2.94311	0.97141	0.93852
Field 3			
24	(σ_1_ − σ_3_)/2 = ((σ_1_ + σ_3_)/2)·0.613544 + 3.02367	0.88786	0.77821
Field 4			
13	(σ_1_ − σ_3_)/2 = ((σ_1_ + σ_3_)/2)·0.60719 + 4.02833	0.92837	0.84931
Field 5			
17	(σ_1_ − σ_3_)/2 = ((σ_1_ + σ_3_)/2)·0.600203 + 1.90923	0.91476	0.82590
Field 6			
8	(σ_1_ − σ_3_)/2 = ((σ_1_ + σ_3_)/2)·0.576689 + 4.66864	0.93622	0.85888
Fields 1–6			
94	(σ_1_ − σ_3_)/2 = ((σ_1_ + σ_3_)/2)·0.610676 + 2.28335	0.91788	0.84071

**Table 12:** The results of regression analysis for the dependence of radial pressure (σ_3_) on failure stress (σ_1_).

Number of Samples	Equation	Pearson’s r	R-Square
Field 1			
18	σ_1_ = σ_3_·3.16264 + 22.223	0.82581	0.65749
Field 2			
14	σ_1_ = σ_3_·2.91776 + 16.4585	0.94056	0.85416
Field 3			
24	σ_1_ = σ_3_·2.08586 + 43.6285	0.63471	0.37442
Field 4			
13	σ_1_ = σ_3_·2.68215 + 37.3136	0.76213	0.54274
Field 5			
17	σ_1_ = σ_3_·2.47493 + 30.4871	0.73187	0.50467
Field 6			
8	σ_1_ = σ_3_·2.74518 + 34.6821	0.82007	0.62574
Fields 1–6			
94	σ_1_ = σ_3_·2.51861 + 32.9488	0.72585	0.52148

**Table 13:** Cohesion and internal friction angle of the analysed rock salt.

Origin of Samples	Number of Samples	Regression Analysis	Cohesion c [MPa]	Internal Friction Angle ɸ [°]
Field 1	18	Stress path, Pearson’s r = 0.95353	2.12	38.42
		Critical state, Pearson’s r = 0.82581	6.25	31.30
Field 2	14	Stress path, Pearson’s r = 0.97141	3.46	31.60
		Critical state, Pearson’s r = 0.94056	4.81	29.31
Field 3	24	Stress path, Pearson’s r = 0.88786	3.83	37.85
		Critical state, Pearson’s r = 0.63471	15.10	20.60
Field 4	13	Stress path Pearson’s r = 0.92837	5.07	37.39
		Critical state, Pearson’s r = 0.76213	11.39	27.18
Field 5	17	Stress path, Pearson’s r = 0.91476	2.39	36.88
		Critical state, Pearson’s r = 0.73187	9.69	25.12
Field 6	8	Stress path, Pearson’s r = 0.93622	5.72	35.22
		Critical state, Pearson’s r = 0.82007	10.47	27.77
Fields 1–6	94	Stress path, Pearson’s r = 0.91788	2.88	37.64
		Critical state, Pearson’s r = 0.72585	10.38	25.57

**Table 14:** The average physical and mechanical parameters of rock salt from the Kłodawa salt deposit.

Bulk Density [kg/m^3^]	Porosity [-]	UCS [MPa]	E [MPa]	ν [-]	TS [MPa]	c [MPa]/ɸ [°]
Field 1						
2095	3.06	17.05	2227	0.28	1.06	2.12/38.42
Field 2						
2079	3.77	26.04	2150	0.30	1.17	3.46/31.60
Field 3						
2091	3.53	20.61	2454	0.27	1.07	3.83/37.85
Field 4						
2087	3.95	20.86	1546	0.26	1.52	5.07/37.39
Field 5						
2081	3.82	19.3	2396	0.33	1.04	2.39/36.88
Field 6						
2119	2.01	20.16	2745	0.34	1.34	5.72/35.22
Fields 1–6						
2090	3.45	20.50	2326	0.29	1.12	2.88/37.64
	—Low variation		—Average variation		—Strength variation	

## Data Availability

The original contributions presented in the study are included in the article, further inquiries can be directed to the corresponding author.
